# Supraliminal But Not Subliminal Distracters Bias Working Memory Recall

**DOI:** 10.1037/xhp0000052

**Published:** 2015-04-13

**Authors:** Theresa Wildegger, Nicholas E. Myers, Glyn Humphreys, Anna C. Nobre

**Affiliations:** 1 Department of Experimental Psychology, University of Oxford; 2 Department of Experimental Psychology and Oxford Centre for Human Brain Activity, University of Oxford; 3 Department of Experimental Psychology, University of Oxford; 4 Department of Experimental Psychology and Oxford Centre for Human Brain Activity, University of Oxford

**Keywords:** working memory, subliminal processing, visual cognition, distracter interference, consciousness

## Abstract

Information of which observers are not consciously aware can nevertheless influence perceptual processes. Whether subliminal information might exert an influence on working memory (WM) representations is less clear, and relatively few studies have examined the interactions between subliminal and supraliminal information in WM. We present 3 experiments examining this issue. Experiments 1a and b replicated the finding that orientation stimuli can influence behavior subliminally in a visuomotor priming task. Experiments 2 and 3 used the same orientation stimuli, but participants had to remember a target orientation and report it back by adjusting a probe orientation after a memory delay. Before or after presentation of the target orientation, a subliminal or supraliminal distracter orientation was presented that was either irrelevant for task completion and never had to be reported (Experiment 2), or was relevant for task completion because it had to be reported on some trials (Experiment 3). In both experiments, presentation of a supraliminal distracter influenced WM recall of the target orientation. When the distracter was presented subliminally, however, there was no bias in orientation recall. These results suggest that information stored in WM is protected from influences of subliminal stimuli, while online information processing is modulated by subliminal information.

Subliminal stimuli have been shown to influence processing of subsequent stimuli at a number of levels—from low-level visual priming up to and including semantic priming (Dehaene et al., 1998; Eimer & Schlaghecken, 1998, 2002; Greenwald, Draine, & Abrams, 1996; Neumann & Klotz, 1994). Acceptance of subliminal effects is not universal (cf., Pratte & Rouder, 2009; Reingold, 2004), but there are numerous demonstrations of subliminal influences on response times in priming tasks (Kiefer et al., 2011; Kouider & Dehaene, 2007). For example, Eimer and Schlaghecken (2002) examined the influence of masked prime stimuli—which could not be identified above chance—on responses to a target stimulus and showed that both subliminal and supraliminal prime stimuli influenced participants’ reaction times (RTs) to target stimuli, though interestingly, in different ways. These results were replicated and their interpretation supported by additional control studies (see Eimer & Schlaghecken, 2003 for a review).

Past studies have focused on the effects of subliminal stimuli on responses to stimuli immediately present in the observer’s environment. However, it remains unknown whether subliminal information can influence what becomes consciously available in working memory (WM). WM representations are short-lived representations that are strictly limited in their capacity (Curtis & D’Esposito, 2003; Zhang & Luck, 2008). Theorists have conceptualized WM in different ways. For example, one common conception is that WM depends on attentional selection of stimuli into a limited capacity store (Baddeley, 1986; Bundesen, 1990), which is distinct from perceptual and semantic representations of the stimuli. Alternatively, it has been argued that WM reflects the temporary activation of perceptual and semantic representations of stimuli (Cowan, 1995).

These different theories posit different putative effects of subliminal processing on WM. For example, if WM involves the activation of perceptual and semantic representations of stimuli, then there is little reason to think that WM should be immune from the influence of subliminal information. The same perceptual and semantic representations would be recruited in WM identification tasks that use similar stimuli to tasks in which visuomotor priming has been established. On the contrary, if WM is abstracted from the perceptual and semantic representations used online for object identification, then it may be that stimuli must be supraliminal, entering the limited-capacity WM store, to influence processing.

Silvanto and Soto (2012) examined the influence of a subliminal visual distracter on performance in a WM task. Participants had to retain a target orientation in memory to compare against a probe orientation after a delay period. During the delay period a masked distracter orientation was presented on some trials. Accuracy in the task was significantly reduced when an incongruent distracter was presented during the WM delay period, relative to both congruent and no-distracter conditions, suggesting that distracting subliminal stimuli influence the maintenance and/or retrieval of WM representations.

One important aspect of the Silvanto and Soto study is that they used subjective rather than objective measures of subliminal processing, which may have underestimated the degree of conscious access to the masked stimulus (see Hannula, Simons & Cohen, 2005 for a review). In a control experiment, forced-choice identification was very high (*d*′ > .80), suggesting that the stimuli may have been available to objective report, although they were not available to subjective conscious report. This difficulty can be overcome if objective measures of awareness are taken as well as subjective measures. Whereas subjective measures are good for evaluating an observer’s internal subjective experience of the environment, they might be based not only on sensitivity to stimulus presence but also on the observer’s decision criterion. Observers may indicate no awareness when their perceptual experience of a stimulus simply failed to surpass their criterion for confidently reporting it.

Here we present three experiments testing effects of subliminal and supraliminal stimuli on perceptual and WM reports. In all cases objective and subjective measures of stimulus awareness were used. The first experiment was an adaptation of studies by Eimer & Schlaghecken (1998, 2002) using stimulus parameters matched to the subsequent WM experiments. In Eimer and Schlaghecken (1998), prime and target stimuli were assigned either the same, or opposite, responses (on congruent and incongruent trials). The prime stimulus, when masked, influenced responses to the target: RTs were slower and more errors made following congruent compared to both neutral and incongruent primes. In addition, neural markers of motor preparation (the lateralized readiness potential measured using event-related potentials) were also affected by prime-target congruency. Specifically, prime stimuli first elicited their corresponding response but this initial activation then reversed, meaning an incongruent response was activated for congruent trials while the congruent response was activated for incongruent trials. The authors argued that the effects reflect inhibition of the response initially activated by the prime, when the prime is masked (Eimer & Schlaghecken, 1998, 2002). We aimed to replicate the behavioral results using the stimuli employed in the other experiments here, which targeted WM rather than perceptual report. Two versions of the experiment were conducted, using blocked (Experiment 1a) and trial-by-trial (Experiment 1b) measures of subjective and objective awareness.

Experiment 2 was a WM task in which we asked participants to remember the orientation of one of two sequentially presented gratings for later recall. One orientation was a target and the other a distracter. We varied the visibility of the stimuli, rendering one subliminal in some conditions (based on objective measures) and supraliminal in others. We examined whether subliminal information could influence WM representations. Experiment 3 was a replication and extension of Experiment 2. We assessed whether any influence of subliminal information might depend on its task relevance by occasionally probing the subliminal stimuli so that they became task-relevant.

## General Method

### Participants

All experimental protocols were reviewed and approved by the Central University Research Ethics Committee of the University of Oxford. A total of 85 volunteers participated in the experiments: 31 males and 54 females, ranging from 18 to 34 years of age. All participants had normal or corrected-to-normal vision and were naïve to the purpose of the experiment. No participants participated in more than one experiment. Participants gave informed consent before taking part, and received course credits or financial compensation (£8 per hour) for taking part.

### Experimental Procedures

**Figure 1 fig1:**
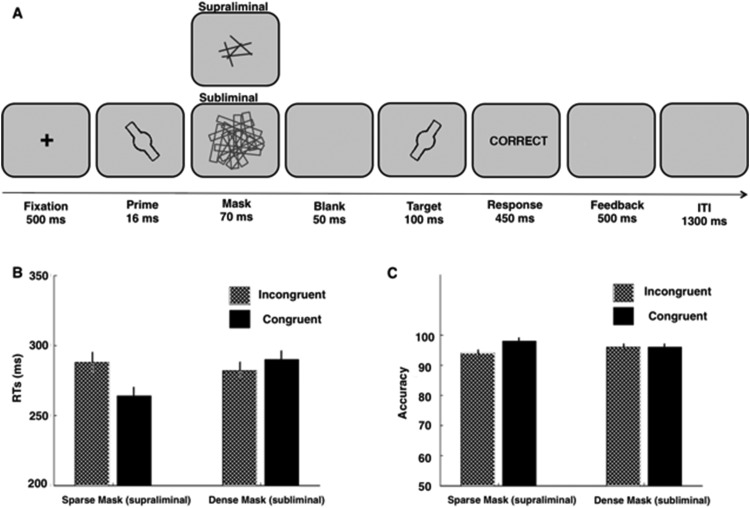
Design for Experiments 1a and 1b, and results for Experiment 1a (1b in supplemental) of the visual priming task. (A) A central fixation cross was presented for 500 ms followed by a 16-ms prime stimulus, and then a 70-ms dense or sparse backward mask. After a 50-ms mask-target interval, a target stimulus (congruent or incongruent with the prime stimulus) appeared for 100 ms. Participants had 450 ms to indicate the orientation (left or right) of the target stimulus and were given 500 ms feedback on their performance. After a 1,300-ms intertrial interval the next trial began. (B) Median RTs to probes following congruent and incongruent prime stimuli followed by a dense or sparse mask (subliminal and supraliminal, respectively) in Experiment 1a. Error bars reflect ± 1 standard error. (C) Mean accuracy to probes following congruent and incongruent prime stimuli followed by a dense or sparse mask (subliminal and supraliminal, respectively) in Experiment 1a. Error bars reflect ±1 standard error.

Experiments were prepared and presented using the Psychophysics Toolbox in Matlab (Brainard, 1997; Kleiner, Brainard, & Pelli, 2007; Pelli, 1997;). The stimuli were presented on a 23-in. LED display with a spatial resolution of 1,920 × 1,080 and a vertical refresh rate of 120 Hz. Stimuli were presented in dark gray (14.5 cd/m^2^) at the center of the screen against a light gray background (25.7 cd/m^2^). Orientation stimuli used throughout the study consisted of the contours of an oriented bar (2.5° visual angle in length and 0.5° visual angle in width) superimposed on a circle (1° visual angle in diameter) forming the outline of a “UFO” shape (Figure 1A). The probe orientation stimulus was identical to the distracter and target orientation stimulus except for its color (light green, 21.3 cd/m^2^). This change in color made the probe item easily distinguishable from the to-be-remembered orientation stimuli in the sequence. Pattern masks were created on each trial by overlaying the contours of 20 black (1.7 cd/m^2^) randomly oriented bars, each with a randomly determined offset of maximally 1° visual angle from the center of the screen (max. length and width: 3.5° visual angle). Thus, the exact appearance of the mask varied from trial to trial. The bars used in creating the mask were identical to those used to create the “UFO” stimuli.[Fig-anchor fig1]

## Experiment 1a: Influence of Subliminal Stimuli in a Visual Priming Task

In Experiment 1 we aimed to demonstrate that distracting orientation stimuli, which remain subliminal by objective criteria, influence orientation judgments about an orientation probe stimulus. To this end we set out to replicate priming effects previously reported by Eimer and Schlaghecken (1998, 2002), but here using orientation, rather than arrow, stimuli. Eimer and Schlaghecken (1998, 2002) reported opposite effects on RTs following subliminal and supraliminal primes. Specifically, congruent supraliminal primes speeded RTs to the probe. When primes were rendered subliminal this effect reversed, and RTs were faster following incongruent compared to congruent primes. We predicted the same pattern of effects for our experiment.

### Method

#### Participants

Twenty volunteers took part (7 male, 18–30 years, one left-handed).

#### Stimuli

Following the design of Eimer & Schlaghecken (1998, 2002), two orientations were used throughout the task: left (120°) and right (60°). Two different types of masks were employed: a dense and a sparse mask. The dense mask was created as described in the General Method section. The sparse mask consisted of only five randomly overlapping horizontal, vertical, and oblique lines (length = 2.5° visual angle, width = 0.12° visual angle) each with a randomly determined offset of maximally 0.36° visual angle from the center (see Figure 1A for an example of stimuli and masks used). The dense mask was intended to render stimuli subliminal, while the sparse mask was intended to preserve stimulus visibility (Eimer & Schlaghecken, 1998, 2002).

#### Task and procedure

The experiment comprised two tasks: A visual priming task and a prime-identification task. The purpose of the priming task was to examine whether the prime orientation stimuli could influence RTs to the subsequent probe orientation stimuli; in other words, whether these kinds of subliminal stimuli are sufficient to influence behavior. The purpose of the identification task was to ensure that prime stimuli were rendered subliminal by the dense mask but remained supraliminal with the sparse mask. Figure 1A provides a graphical summary of the priming task. Each trial began with the presentation of a central fixation cross (500-ms duration). A prime stimulus (16-ms duration) was immediately followed by a mask (70-ms duration), then followed by a 50-ms blank screen, and finally by the target stimulus (100-ms duration). Participants responded according to the orientation of the target (left or right). Visual feedback (“correct,” “incorrect,” “too slow”) was shown for 500 ms after a response was made or after 450 ms (whichever occurred first). The intertrial interval was 1,300 ms. Prime and target orientations were either the same, or opposite (i.e., congruent or incongruent). In other words, if the prime stimulus was 60° the target stimulus could be 60° or 120°. Both congruency conditions were equiprobable and randomized within each block. Mask type was varied between blocks, and block order was counterbalanced across participants. Participants were instructed to respond as quickly as possible without sacrificing accuracy, using their left and right index fingers for “left” and “right” orientations, respectively.

The priming task consisted of a factorial design with two within-subject factors: probe (same, different) and visibility (supraliminal, subliminal). Participants completed eight blocks of 40 trials each (320 trials in total, 80 trials per condition). Four blocks were run using the dense mask (160 trials), the other four with the sparse mask (160 trials). Blocks alternated between dense-mask blocks and sparse-mask blocks, and the starting block-type was counterbalanced across participants. Breaks were given after every block. Participants completed a practice block of 40 trials before they started the priming task.

**Figure 2 fig2:**
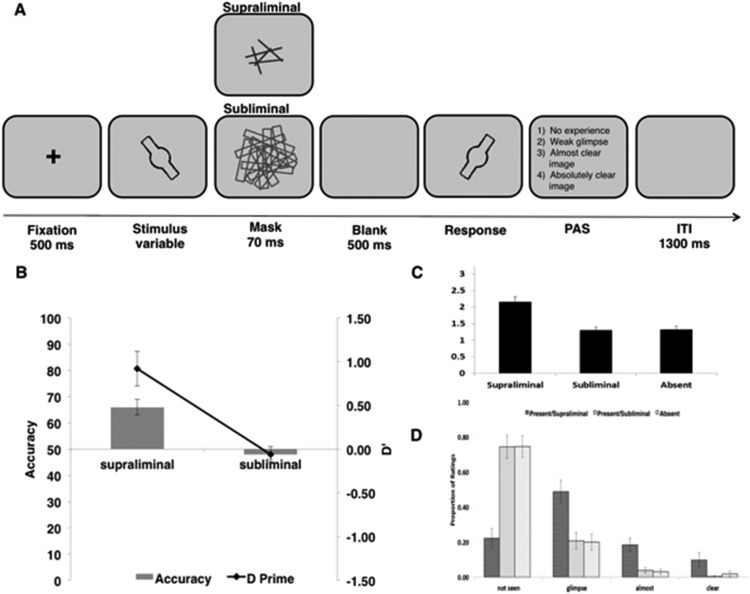
Design of the visual identification task used in Experiments 1a and 1b, and results of the visual identification task in Experiment 1a. (A) A central fixation cross was presented for 500 ms followed by a 16-ms prime stimulus (present on 50% of trials, blank shown on other 50% of trials), and then a 70-ms dense or sparse backward mask. After a 50-ms mask-probe interval, a probe stimulus (congruent or incongruent with the prime stimulus) appeared for 100 ms. Participants first completed a forced-choice discrimination task indicating whether the probe was of the same or different orientation as the stimulus. Next, participants rated their subjective awareness using the PAS (Ramsøy & Overgaard, 2004). After a 1,300 ms intertrial interval the next trial began. (B) Accuracy and *d*′ in the forced-choice identification task for the dense and sparse mask condition separately. (C) Mean awareness ratings for the sparse-mask, dense-mask, and stimulus-absent conditions separately. (D) Proportion of different awareness ratings in the identification task for the three conditions. Error bars reflect ±1 standard error.

A visual identification task was used to obtain objective and subjective measures of awareness for stimuli followed by dense and sparse masks (see Figure 2A). The same stimuli and masks as in the priming task were used. Participants were presented with a stimulus in half the trials to assess observers’ ability to discriminate between stimulus presence and absence as well as their subjective awareness of the stimulus. When present, the stimulus appeared for 16 ms and was followed by a 70-ms presentation of either the dense or sparse mask. Mask density was varied between blocks, and block order was counterbalanced across participants. Following a delay of 500 ms, a probe stimulus was shown (congruent or incongruent with equal probability). In the other half of the trials, when no stimulus appeared, the procedure was the same except that a blank display instead of a stimulus was shown before the mask. Participants first completed a forced-choice discrimination task indicating whether the probe was of the same or different orientation as the stimulus. Next, participants rated their subjective awareness using the Perceptual Awareness Scale (PAS, Ramsøy & Overgaard, 2004). This scale consists of four response options defined as follows: 1 (*stimulus not seen*); 2 (*weak glimpse: something was there but I do not know its orientation*); 3 (*almost clear image: I think I know the orientation*); and 4 (*clear image*). Participants were instructed on how to use this scale before the first session of the identification task. It was emphasized that ratings are to be made introspectively, relying on visual experience (Ramsøy & Overgaard, 2004). A shortened version of the four ratings was spelled out on the screen at the end of each trial before ratings were made [1 (*stimulus not seen*), 2 (*weak glimpse*), 3 (*almost clear image*), 4 (*clear image*)]. Participants responded by pressing the corresponding number on the keyboard.[Fig-anchor fig2]

The identification task consisted of the same 2 × 2 (probe: same, different; visibility: supraliminal, subliminal) design as the priming task with the additional factor of prime presence (present, absent). Thus, when no prime was present, probe orientation cannot be described as “same” or “different” relative to the probe, but instead was randomly determined to be either 120° or 60°. Participants completed four blocks of 50 trials each (200 trials in total). Thus, there were a total of 25 trials per target-present condition. Two blocks were run with the dense mask (100 trials). The other two were run with the sparse mask. Blocks alternated between dense-mask and sparse-mask blocks, and the starting block-type was counterbalanced across participants.

Participants completed one experimental session lasting approximately 45 min. All participants first completed the priming task and then completed the identification task.

#### Analysis

For the priming task, we analyzed median RTs and accuracy. For RTs, we calculated the median rather than the mean since it is more robust to outliers. Only correct responses were included in the RT analysis.

Performance in the identification task was measured in levels of accuracy, *d*′ in stimulus-present trials, and subjective ratings of awareness for both stimulus-present and stimulus-absent trials. D’ was calculated from the hit rate and false-alarm rate using the following equation (Macmillan & Creelman, 1991):

$$d^{\prime }=z(\text{Hit})-z(\text{FA}).$$ 1

Hit rate was defined as the probability that the participant responded “different” when the probe orientation was different, and the false-alarm rate was defined as the probability that the participant responded “different” when the probe orientation was the same.

In the case of hit rates of 1 and/or false alarm rates of 0, values were adjusted using the following equation (Macmillan & Creelman, 1991, p. 8):

$$\text{Adjusted}\;\text{Hit}=1-\frac{1}{2\text{*Nr of Trials}}.$$ 2

$$\text{Adjusted}\;\text{FA}=\frac{1}{2\text{*Nr of Trials}}.$$ 3

### Results

#### Visual priming task

Figure 1B and 1C show RTs and accuracy for each condition. We replicated the expected pattern of speeded RTs following congruent supraliminal primes and slowed RTs following congruent subliminal primes (Eimer & Schlaghecken, 1998, 2002). This was reflected in a highly significant mask-by-congruency interaction (*F*(1, 19) = 45.32, *p* < .001, η^2^ = .71, JZS Bayes factor: > 500). To examine this interaction further, Bonferroni-corrected paired *t* tests were conducted comparing congruent and incongruent trials for each mask condition separately. The difference between responses in congruent and incongruent trials was significant for both the sparse and dense masks. In the sparse-mask condition, responses were significantly faster following congruent primes compared to incongruent primes (*M*_Congruent_ = 261 ms ± 6, *M*_Incongruent_ = 288 ms ± 8; *t*(19) = 4.76, *p* < .001, *d* = 1.06, JZS Bayes factor: 211). In the dense-mask condition, this effect reversed, and responses were significantly faster following incongruent primes compared to congruent primes (*M*_Congruent_ = 289 ms ± 6, *M*_Incongruent_ = 280 ms ± 7; *t*(19) = −2.43, *p* = .025, *d* = 0.54, JZS Bayes factor: 2.40). The main effects of probe congruency and mask were also significant (*F*(1, 19) = 4.48, *p* < .05, η^2^ = .19, JZS Bayes factor: 1.01, and *F*(1, 19) = 5.04, *p* < .05, η^2^ = .21, JZS Bayes factor: 1.62, respectively). The same pattern of results was observed with mean, rather than median, RTs (mask-by-congruency interaction: *F*(1, 19) = 51.23, *p* < .001, η^2^ = .73, JZS Bayes factor: > 500; main effect of congruency: *F*(1, 19) = 5.12, *p* < .05, η^*2*^ = .21, JZS Bayes factor: 0.96; main effect of visibility: *F*(1, 19) = 4.96, *p* < .05, η^2^ = .207, JZS Bayes factor: 2.78; *t* tests dense-mask condition: *M*_Congruent_ = 290 ms ± 6, *M*_Incongruent_ = 282 ms ± 7; *t*(19) = −2.36, *p* = .029, *d* = 0.53, JZS Bayes factor: 2.13; sparse-mask condition: *M*_Congruent_ = 264 ms ± 6, *M*_Incongruent_ = 288 ms ± 8; *t*(19) = 4.98, *p* < .0001, *d* = 1.11, JZS Bayes factor: 328).

For accuracy, there was a significant mask-by-congruency interaction (*F*(1, 19) = 10.26, *p* < .01, η^2^ = .35, JZS Bayes factor: 30.16). Bonferroni-corrected paired-samples *t* tests, comparing congruent and incongruent trials for each mask condition separately, revealed that accuracy was significantly better following congruent primes compared to incongruent primes in the sparse mask condition (*M*_Congruent_ = 98% ± .6, *M*_Incongruent_ = 94% ± .8, *t*(19) = −4.08, *p* < .01, *d* = 0.91, JZS Bayes factor: 54). In the dense-mask condition, there was no difference in accuracy between congruent and incongruent prime trials (*M*_Congruent_ = 96% ± .7, *M*_Incongruent_ = 96% ± .7, *t*(19) = −.193, *ns,* JZS Bayes factor in favor of the null: 4.23). The main effect of congruency was also significant (*F*(1, 19) = 9.29, *p* < .01, η^2^ = .33, JZS Bayes factor: 10.84).

#### Prime-identification task with blocked design

Data from one participant in the prime-identification task were not available because the data files were mistakenly overwritten.

##### Accuracy and d′

Figure 2B shows accuracy and *d*′ in the forced-choice discrimination task for the stimulus present/supraliminal and present/subliminal condition separately. A paired-samples *t* test compared accuracy in stimulus-present conditions using dense versus sparse masks. There was a significant difference between the conditions reflecting that participants performed significantly better in the sparse mask condition than in the dense-mask condition, *t*(18) = - 4.78, *p* < .001, JZS Bayes factor: 196, *d* = 1.50, *M*_sparse_ = 66% ± 3, *M*_dense_ = 48% ± 2. Furthermore, performance in the dense-mask condition was not significantly better than chance (*t*(18) = −.722, *ns,* JZS Bayes factor in favor of the null: 3.34).

Similarly, *d*′ in the sparse-mask and dense-mask conditions differed significantly, *t*(18) = 4.64, *p* < .001, *d* = 1.48, JZS Bayes factor: 149. Participants performed significantly better in the sparse-mask than in the dense-mask condition (*M*_sparse_ = 0.92 ± 0.2, *M*_dense_ = - .06 ± .09). Importantly, *d*′ in the dense-mask condition was not significantly different from 0 (*t*(18) = .476, *ns,* JZS Bayes factor in favor of the null: 3.80). These results show that participants were not better than chance at detecting and discriminating orientation in the dense-mask condition. However, stimuli followed by the sparse mask were reliably detected and their orientation discriminated.

##### Subjective ratings

Figure 2C and 2D show the mean awareness ratings and proportion of different ratings for each condition separately. A Wilcoxon signed-ranks test comparing average awareness ratings in the sparse-mask, dense-mask, and absent conditions indicated that there was no difference in ratings between the absent (*M* = 1.32 ± 0.10) and dense-mask condition (*M* = 1.30 ± 0.09, *Z* = −0.26, *ns*; see Figure 2). However, there was a significant difference between the average awareness ratings in the sparse-mask (*M* = 2.16 ± 0.15) and absent condition (*Z* = −3.82, *p* < .001), and the sparse-mask and dense-mask conditions (*Z* = −3.82, *p* < .001, respectively).

To examine what was driving these differences, we calculated the proportions of each rating for each participant separately, and then ran eight separate Wilcoxon signed-ranks test comparing the proportions of ratings between the absent- and sparse-mask condition, and the dense-mask and sparse-mask condition for each rating option separately. “Not Seen” ratings occurred significantly more often in the dense-mask and absent condition compared to the sparse-mask condition (*Z* = −3.77, *p* < .001 and *Z* = −3.82, *p* < .001, respectively). Conversely, “Weak Glimpse,” “Almost Clear” and “Absolutely Clear” ratings were made significantly more often in the sparse-mask condition compared to the absent and dense-mask condition (all *p*’s < 0.01).

Together, these results suggest that participants were unable to discriminate stimulus orientations on dense-mask trials, and unable to differentiate reliably between dense-mask and stimulus-absent trials. Stimulus orientation on sparse-mask trials, however, was reliably discriminated.

### Discussion

The results replicated previous findings (Eimer & Schlaghecken, 1998, 2002), showing that both subliminal and supraliminal orientation stimuli can influence discrimination responses to a probe stimulus. Participants were faster and more accurate to respond to an orientation stimulus when a supraliminal prime of the same orientation preceded the target stimulus. When the prime stimulus was presented subliminally, this effect reversed and participants were faster to respond to an orientation stimulus when the prime orientation was different to the target orientation. In contrast to supraliminal primes, there was no effect of subliminal primes on accuracy. These results show that both orientation stimuli (as used in this experiment), and arrow stimuli (as used in Eimer & Schlaghecken, 1998, 2002), can influence behavior when presented subliminally.

## Experiment 1b: Replication of Visual Priming With Alternative Assessment of Prime Awareness

In Experiment 1a we used a blocked design to test for objective awareness of stimuli in the prime-identification task. However, it has been shown that blocked designs can underestimate prime awareness (Pratte & Rouder, 2009), since participants may become disengaged and unmotivated in difficult, subliminal blocks. When subliminal and supraliminal trials are mixed together, relatively easy and relatively difficult trials vary randomly keeping participants engaged with the task. Correspondingly, estimates for chance identification of subliminal primes are different in mixed compared to blocked identification tasks. To ensure that the effects of subliminal primes in Experiment 1a were not due to residual awareness of the stimuli, we tested another 20 participants on the same visuomotor priming task followed by a prime-identification task in which sparse and dense mask conditions were randomly intermixed on a trial-by-trial basis.

### Method

#### Participants

Twenty volunteers took part (9 male, 18–34 years old, one left-handed).

#### Stimuli, task, procedure, and analysis

The design, parameters, and analysis of the visual priming task were identical to Experiment 1a. Experiment 1b was, therefore, a straight replication of the previous experiment on an independent set of participants. The prime-identification task was also equivalent, with the exception that the dense-mask and sparse-mask trials were intermixed randomly, on a trial-by-trial basis, within a single testing block.

### Results

#### Visual priming task

We replicated the pattern of results observed in Experiment 1a. A significant mask-by-congruency interaction in median RTs (*F*(1, 19) = 42.06, *p* < .001, η^2^ = .69, JZS Bayes factor: >500) showed significant and opposite effects of prime congruency in the sparse-mask (*M*_Congruent_ = 246 ms ± 7, *M*_Incongruent_ = 266 ms ± 7; *t*(19) = 4.64, *p* < .001, *d* = 1.04, JZS Bayes factor: 166) and dense-mask conditions (*M*_Congruent_ = 276 ms ± 7, *M*_Incongruent_ = 270 ms ± 8; *t*(19) = −2.02, *p* = .058, *d* = 0.45, JZS Bayes factor: 1.25). As before, main effects of congruency and visibility were also significant (*F*(1, 19) = 4.44, *p* = .048, η^2^ = .181, JZS Bayes factor: 0.65 and *F*(1, 19) = 14.94, *p* = .001, η^2^ = .44, JZS Bayes factor: > 500, respectively). The same pattern of results was observed with mean RTs (mask-by-congruency interaction: *F*(1, 19) = 43.53, *p* < .001, η^2^ = .56, JZS Bayes factor: >500; main effect of congruency: *F*(1, 19) = 4.41, *p* = .049, η^2^ = .188, JZS Bayes factor: 0.65; main effect of visibility: *F*(1, 19) = 13.57, *p* < .01, η^2^ = .42; JZS Bayes factor: >500, sparse-mask condition: *M*_Congruent_ = 251 ms ± 7, *M*_Incongruent_ = 268 ms ± 6; *t*(19) = 4.39, *p* < .001, *d* = 0.98, JZS Bayes factor: 92; dense-mask condition: *M*_Congruent_ = 277 ms ± 7, *M*_Incongruent_ = 272 ms ± 6; *t*(19) = −2.63, *p* = .016, *d* = 0.58, JZS Bayes factor: 3.36).

Similarly, we replicated our findings in accuracy. A significant mask-by-congruency interaction (*F*(1, 19) = 34.49, *p* < .001, η^2^ = .65, JZS Bayes factor: 116) revealed significantly better accuracy following congruent primes compared to incongruent primes in the sparse-mask condition (*M*_Congruent_ = 97% ± .7, *M*_Incongruent_ = 92% ± 1.4; *t*(19) = −4.57, *p* < .01, *d* = 1.02, JZS Bayes factor: 144) and no difference in the dense-mask condition (*M*_Congruent_ = 95% ± 1.6, *M*_Incongruent_ = 95% ± 1.1; *t*(19) = 0.25, *ns,* JZS Bayes factor in favor of the null: 4.18). The main effect of congruency was also significant (*F*(1, 19) = 7.70, *p* = .012, η^2^ = .29, JZS Bayes factor: 10.69).

Analysis of performance in the prime-identification task revealed that five participants performed better than chance at identifying the subliminal prime (see below). Importantly, when we excluded those participants from analysis, we observed the same pattern of results. A significant mask-by-congruency interaction in median RT data (*F*(1, 14) = 35.47, *p* < .001, η^2^ = .72, JZS Bayes factor: >500) showed opposite effects of congruency in the sparse-mask (*M*_Congruent_ = 244 ms ± 8, *M*_Incongruent_ = 259 ms ± 8; *t*(14) = 3.17, *p* = .007, *d* = 0.81, JZS Bayes factor: 7.65) and the dense-mask (*M*_Congruent_ = 273 ms ± 8, *M*_Incongruent_ = 267 ms ± 9; *t*(14) = −2.10, *p* = .054, *d* = 0.54, JZS Bayes factor: 1.44) conditions. A significant mask-by-congruency interaction in the accuracy data (*F*(1, 14) = 19.78, *p* = .001, η^2^ = .59, JZS Bayes factor: 5.54) was driven by a significant effect of congruency in the sparse-mask condition (*M*_Congruent_ = 97% ± .9, *M*_Incongruent_ = 92% ± 1.7; *t*(14) = −3.32, *p* < .01, *d* = 0.86, JZS Bayes factor: 9.80) but not in the dense-mask condition (*M*_Congruent_ = 94% ± 1.3, *M*_Incongruent_ = 94% ± 2.1; *t*(14) = −0.19, *ns,* JZS Bayes factor in favor of the null: 3.75).

#### Prime-identification task

Performance in the prime-identification task showed that, at the group level, participants were not better than chance at detecting and discriminating orientation in the dense-mask condition (*t*(19) = −.39, *ns*). In the sparse-mask condition, they reliably detected the stimuli and discriminated their orientation. However, binomial tests applied to responses at the individual subject level (alpha level at .05) indicated that five participants performed significantly better than chance. These five participants showed residual ability to differentiate between stimulus-present and stimulus-absent trials to some extent (see supplementary materials for more details).

### Discussion

In Experiment 1b we used a mixed design to assess prime identification. This revealed some awareness for the prime stimuli in a small number of participants in our sample. This highlights the importance of considering motivational factors when assessing observers’ awareness in a subliminal prime-identification task (Pratte & Rouder, 2009). Precautions must be taken to ensure that observers stay engaged with the task, for example, by mixing subliminal with supraliminal trials. Most importantly, however, we replicated our main findings of subliminal priming in the visuomotor priming task. The replication of the visuomotor priming effects (Eimer & Schlaghecken, 1998, 2002) in two independent sets of participants provides reassurance about their reliability, even though the magnitude of the effects and the Bayes factors in each case are modest. Under the Bayesian framework, it is possible to combine data sets and as one adds more data one is simply adding more evidence to prior (however vague) knowledge (Dienes, 2011). Capitalizing on the identical design and procedures of our two priming tasks, we pooled the data across the two experiments (without excluding any participants) and computed the JZS Bayes factor on the comparison of median (mean) RTs and accuracy between congruent and incongruent primes in the dense-mask condition. For the combined median RTs (*N* = 40; incongruent = 275 ms; congruent = 283 ms; *t* = −3.18), the JZS Bayes factor = 11.97; and for combined mean RTs (*N* = 40; incongruent = 277 ms; congruent = 284 ms; *t* = −3.42), the JZS Bayes factor = 21.54. That is, the data strongly favor the alternative over the null hypothesis, suggesting that subliminal prime stimuli influence behavior response times in our task. When the five participants from Experiment 1b who showed residual awareness were excluded, we observed similar values (median RTs = 11.3; mean RTs = 23.65).

## Experiment 2: Influence of Task-Irrelevant Subliminal Versus Supraliminal Stimuli on WM

Having replicated the finding that subliminal stimuli can influence visuomotor processes in a priming task, we aimed to examine whether subliminal stimuli can influence stimuli that become consciously available in WM representations. Participants were presented with a sequence of two orientation stimuli—one of which was followed by a mask. Participants recalled the orientation of the unmasked target stimulus. The masked stimulus acted as a distracter. On half of the trials, the distracter stimulus subliminal and on the other half it was supraliminal. We examined whether recalling an orientation from WM can be influenced by a distracter orientation, and to test how this varies with changes in the observer’s awareness of the distracter orientation. In line with the visuomotor priming design by Eimer and Schlaghecken (1998, 2002), we were mainly interested in examining the influence of a distracter before encoding, rather than during the delay period.

### Method

#### Participants

Twenty-four new volunteers took part in the experiment (5 male, 18–30 years old, all right-handed). Two additional participants started but did not complete all sessions of the experiment. Their data were not included in the analysis.

#### Stimuli

The orientations of target, distracter, and probe stimuli were randomly determined on each trial and ranged from 0° to 180°. Distracter duration and presentation was varied to yield three conditions occurring in equal proportions: distracter absent, supraliminal distracter present (presented for 200 ms), and subliminal distracter present (duration individually determined, M = 36 ms ± 7; see below for further details). When the distracter was absent, a blank image was shown for either the duration used in the supraliminal or the subliminal condition (randomly determined on each trial). The duration of the mask was 70 ms.

#### Task

**Figure 3 fig3:**
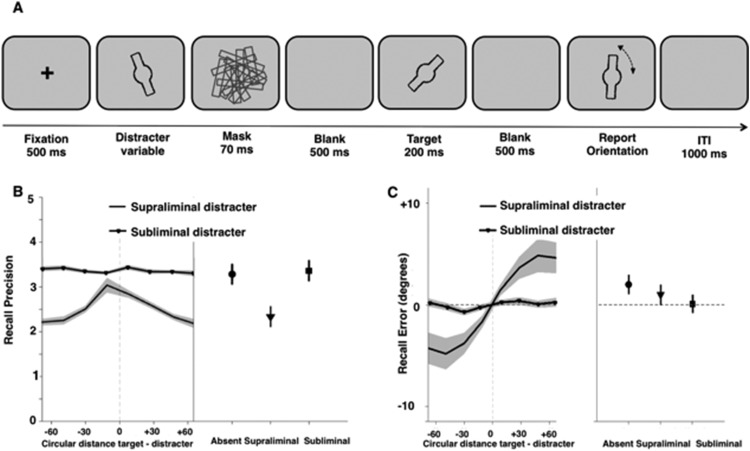
Design and results of the WM task used in Experiment 2. (A) A central fixation cross was presented for 500 ms followed by either the target or distracter orientation stimulus (distracter first on 80% of trials). Participants were instructed to report the orientation of the unmasked stimulus. Thus, the target was always unmasked and the distracter was always immediately followed by a mask. There was a 500-ms delay between offset of the first orientation stimulus (or the subsequent mask if the first orientation stimulus was the distracter) and onset of the second orientation stimulus. After another 500-ms delay, participants reported the target orientation by rotating a probe stimulus to the remembered target orientation by moving the computer mouse. (B) Mean recall precision (1 *SD*) for the distracter conditions as a function of target-distracter similarity (left) and mean overall recall precision for the two conditions (*N* = 23) in Experiment 2. For plotting purposes, the data for this and all similar figures depicting response error and recall precision were smoothed by using eight overlapping bins in the analysis where adjacent bins overlap by 50% and each bin contains 25% of the overall data. Shaded areas and error bars reflect ±1 standard error. (C) Mean recall error for the supraliminal and subliminal conditions as a function of target-distracter similarity (left) and mean overall recall error for absent, supraliminal, and subliminal conditions (*N* = 23) in Experiment 2.

The experiment comprised two tasks: A WM task (see Figure 3A) and a visual identification task (see supplementary Figure 1A). The purpose of the WM task was to examine whether the orientation of the masked distracter stimulus could influence encoding of the target orientation. The purpose of the identification task was to ensure the mask rendered the distracting stimulus indiscriminable in the subliminal condition.[Fig-anchor fig3]

In the WM task, participants were instructed to report the orientation of the unmasked stimulus. Each WM trial began with the presentation of a central fixation cross for 500 ms followed by either the target (unmasked) or distracter (masked) orientation stimulus. Since we were primarily interested on testing the effect of subliminal and supraliminal stimuli on WM encoding, the proportion of trials in which the distracter stimulus appeared first was larger (80% of the trials). To ensure that participants attended to all stimuli in the sequence, the distracter was presented after the target on the remaining 20% of the trials. There was a 500-ms delay between offset of the first orientation stimulus (or the subsequent mask if the first orientation stimulus was the distracter) and onset of the second orientation stimulus. After another 500-ms delay after the second orientation stimulus or mask, participants reported the target orientation by rotating a probe stimulus to the remembered target orientation by moving the computer mouse.

The prime-identification task was adapted from the task used in Experiment 1a (see supplementary materials section for details).

#### Design and procedure

Participants completed two sessions on two separate days; the first session lasted approximately 2 hr and the second session lasted approximately 90 min. Before the beginning of the first session, participants completed a staircase procedure to set the duration of stimulus presentation for the subliminal condition. After the staircase procedure, participants completed a practice session of 50 trials in which they received feedback after every trial. The participants then started the WM task. In each session, participants completed both the WM and the identification task, always starting with the WM task. The WM task consisted of a factorial design with the variables “distracter” (absent, supraliminal, subliminal) and “stimulus order” (target first, distracter first). The different trial types were randomly intermixed within a block. Only trials in which the distracter appeared first were used in the analysis.

Overall, participants completed a total of 1,200 trials (24 blocks of 50 trials) of the WM task, resulting in a total of 320 trials in each condition in which the distracter appeared before the target, and 80 trials in each condition in which the distracter appeared after the target. The first 600 trials were completed in the first session, the second 600 trials in the second session. Participants completed a total of 400 trials (four blocks of 100 trials) of the identification task, resulting in a total of 25 trials per condition. The prime-identification task was completed after every sixth block of the WM task (every 300 trials).

#### Staircase procedure

The distracter duration in the subliminal condition was determined before the start of the experiment using a staircase procedure. The staircase task used the same stimulus sequence as the identification task, but without subjective ratings (see supplementary materials). Participants completed 60 trials of the staircase task. Stimuli and masks were identical to those in the WM task. Each trial started with the presentation of an orientation stimulus, followed by a 70-ms mask, and then by a delay of 500 ms, and finally the presentation of a probe stimulus. Participants indicated whether the probe stimulus was identical or rotated by 90° relative to the first orientation. Probe stimuli were of the same orientation in half the trials. We used a one-up/one-down staircase to derive the presentation duration of the first orientation that lead to 50% accuracy. The staircase started with a presentation duration of 50 ms, and dynamically adapted the duration in log steps based on participants’ accuracy every 20 trials. Participants received feedback at the end of each trial in the form of a written word (“correct, “incorrect”) presented centrally on the screen. The staircase procedure was repeated if necessary until participants reliably performed at chance level.

#### Analysis

##### Identification task

Performance in the identification task was measured as described in Experiment 1.

##### Working-memory task

Only trials in which the distracter appeared first were included in the analysis. For each trial, the reported orientation was collected and measured against the orientation of the target stimulus to derive recall error and recall precision. Recall error was defined as the angular deviation between the target orientation and the reported orientation. Recall precision measured the trial-to-trial variability in the response error, and was calculated as the reciprocal of the standard deviation (*SD*) of errors across trials. Since orientation space is circular, we used Fisher’s definition of *SD* for circular data (Fisher, 1993), and subtracted the value expected by chance so that a precision value of zero corresponds to chance performance. In a one-item WM task, recall precision is estimated around 3.5 rad^−1^, and this value decreases as the number of items is increased (Bays, Catalao, & Husain, 2009). First, we compared overall precision in the three conditions. Then, to examine whether, and how, presentation of a distracter orientation had an effect on information stored in WM we estimated recall precision and recall error as a function of target-distracter similarity—that is, the angular difference between the target and distracter orientations. Data from each participant were sorted into eight equally sized nonoverlapping bins (*n* ≈ 40 trials per bin per subject), ranging from −90° to + 90° angular deviation. Recall precision and error were then calculated separately for each subject and condition for each of the eight bins.

Hypotheses regarding the effects of experimental parameters on recall precision and recall error were tested using analysis of variance (ANOVA) and *t* tests.

### Results

#### Working-memory task

##### Recall precision

Overall precision was significantly different between the subliminal-distracter, supraliminal-distracter, and distracter-absent conditions (*F*(2, 46) = 28.70, *p* < .001, η^2^ = 1.11, JZS Bayes factor: >500, see Figure 3), reflecting that precision in the supraliminal-distracter condition was significantly worse than in both the subliminal-distracter and the distracter-absent conditions (*M*_Supraliminal_ = 2.34 rad^−1^ ± 0.221, *M*_Subliminal_ = 3.36 rad^−1^ ± 0.228, and M_Absent_ = 3.29 rad^−1^ ± 0.226, respectively; *t*(23) = −5.75, *p* = < .001, *d* = 1.17, JZS Bayes factor: >500 and *t*(23) = −5.34, *p* = < .001, *d* = 1.09, JZS Bayes factor: >500). Recall precision in the subliminal-distracter and distracter-absent conditions were not different (*t*(23) = 1.124, *ns,* JZS Bayes factor in favor for the null: 2.65).

To compare the effects of subliminal versus supraliminal stimuli on WM encoding, we used 2 × 8 repeated-measures ANOVAs with the variables “visibility” (supraliminal, subliminal) and “target-distracter similarity” (8 nonoverlapping bins ranging from −90° to + 90° angular deviation) on the recall precision and recall error data. Figure 3 shows the recall precision with which participants recalled the target’s orientation as a function of target-distracter similarity for each visibility condition separately. There was a significant interaction between visibility and target-distracter similarity (*F*(7, 161) = 4.28, *p* < .001, η^2^ = .157, JZS Bayes factor: >500). To examine this interaction further, two separate one-way repeated-measures ANOVAs assessed the effect of target-distracter similarity for each condition separately. There was a significant effect of target-distracter similarity in the supraliminal (*F*(7, 161) = 6.00, *p* < .001, JZS Bayes factor: > 500) but not in the subliminal (*F*(7, 161) = 0.49, *ns,* JZS Bayes factor: 0.02) condition. Specifically, the more similar the orientations of the distracter and the target were, the better participants’ recall precision tended to be (see Figure 3B).

##### Recall error

Figure 3C shows recall error for the target orientation as a function of target-distracter similarity for each visibility condition separately. The mean error in the distracter-absent condition was 0.59° ± 0.45°, which was not significantly different from zero (*t*(23) = 1.34, *ns,* JZS Bayes factor in favor of the null: 2.11). Visual inspection of the graph suggests that recall error varied as a function of target-distracter similarity in the supraliminal but not the subliminal-distracter condition. When the distracter orientation was clockwise to the target orientation (a negative circular distance in Figure 3C) recall error was systematically shifted clockwise (negative), while counterclockwise distracters led to a counterclockwise shift in recall error. Indeed, there was a significant interaction between visibility and target-distracter similarity (*F*(7, 161) = 7.15, *p* < .01, η^2^ = .166, JZS Bayes factor: >500) reflecting that there was a significant effect of target-distracter similarity in the supraliminal (*F*(7, 161) = 8.18, *p* = .001, η^2^ = 1.836, JZS Bayes factor: >500) but not the subliminal (*F*(7, 161) = 1.91, *p* = .104, JZS Bayes factor: 0.43) distracter condition.

Analysis of performance in the identification task revealed that one participant was able to discriminate between stimulus present and absent trials, and their individual subjective ratings mirrored this pattern of performance. Effects were the same when we reran the analysis excluding this one participant.

##### Control analyses

Previous studies using similar WM tasks have shown that a nontrivial proportion of overall error rates can be attributed to participants mistakenly reporting the distracter item, that is, *misbinding* of distracter and target identities (Bays et al., 2009). To ensure that the systematic shift in recall error toward the distracter orientation was not exclusively driven by these misbinding trials, we used a mixture-model approach to model different sources of error contributing to the overall distribution of responses (see Bays et al., 2009 for details) using publicly available Matlab code (available on bayslab.com). The original code returns parameter estimates for the probability of reporting the target (α), the probability of reporting the distracter (β), and the probability of responding randomly (γ), on the basis of the entire dataset. However, to estimate these probabilities the relative weights for target, distracter, and random responses are calculated for each trial separately. We used these trial-wise weights to identify target responses, misbinding trials, and guessing trials. Specifically, trials with larger values for distracter weights, that is, misbinding trials, and guess trials, were excluded from analysis. All misbinding trials had distracter weights of >0.90 while all target-related trials had distracter weights of <.10. When we rerun our main analyses (calculating recall precision and recall error as before) we observed the same effects as reported above, but with a reduction in overall recall error (significant effect of target-distracter similarity in the supraliminal (*F*(7, 161) = 3.24, *p* = .009, η^2^ = 0.864, JZS Bayes factor: 10.48) but not the subliminal (*F*(7, 161) = 1.48, *p* = .211, JZS Bayes factor: 0.17) distracter condition:. Therefore, the systematic shift in recall error toward the distracter orientation was not due to distracter intrusions, but due to a bias in target-related responses.

#### Identification task

Performance in the prime-identification task showed that at the group level participants were not better than chance at performing the discrimination task for the masked stimulus (see supplementary materials for more details).

### Discussion

The results show that supraliminal but not subliminal distracters presented before the target orientation strongly influenced the observers’ subsequent recall based on the WM representation. When distracters were supraliminal, observers’ reports of the target orientation were strongly biased toward the distracter orientation, and the strength of this bias varied with the similarity between distracter and target orientations. The effect represented a systematic bias rather than intrusions from trials on which participants incorrectly reported the orientation of the distracter stimuli (misbinding errors). This occurred despite the fact that the distracter was task-irrelevant and never probed. When presented subliminally, distracters exerted no influence on orientation reports. The Bayesian analysis supports the conclusion that this is a true null effect and not due to low sensitivity of the task design (which would be reflected in a Bayes factor closer to 1 than to 0).

The results, therefore, suggest that subliminal stimuli are unable to influence WM representations that become available for conscious report. However, the fact that the masked stimuli in the current task were consistently task-irrelevant and could be completely ignored may have dampened any influence they may have otherwise exerted. It is well known that task relevance can modulate stimulus processing (Ansorge & Neumann, 2005; Gayet, Van der Stigchel, & Paffen, 2014; Gorgoraptis, Catalao, Bays, & Husain, 2011; Wyart, Nobre, & Summerfield, 2012; Zokaei, Manohar, Husain, & Feredoes, 2014). In particular, task relevance has been shown to mediate the effect of subliminal stimuli in some tasks (Ansorge & Neumann, 2005; Gayet at al., 2014). Gayet et al. (2014) asked participants to complete a peripheral target-detection task with central arrow cues that were either subliminal or supraliminal (randomly intermixed), with the majority of cues being supraliminal. Subliminal cues were never predictive, while supraliminal cues could be nonpredictive or highly predictive (presented as separate experimental conditions on separate days). It was found that subliminal arrow cues only facilitated performance when they were presented among predictive supraliminal cues. This facilitatory effect increased over the course of the experiment, suggesting that the usage of subliminal cues was based on participants learning the predictive value of the supraliminal cue. Subliminal information only appears to be used when it is presented in a context where there is a reason to use it.

Thus, in the next experiment, we changed the task so that the subliminal orientation stimulus was task-relevant. Specifically, participants now had to report the orientation of the masked item on a proportion of trials even when it was presented subliminally. We hypothesized that task relevance would encourage processing of the masked subliminal stimuli, thus allowing them to bias orientation reports on subsequent targets.

## Experiment 3: Influence of Task-Relevant Subliminal Versus Supraliminal Stimuli on WM

### Method

#### Participants

Twenty-one new subjects took part in the experiment (10 male, 18 – 32 years, two left-handed). Two additional participants were tested but their data were not included in the analysis as their task performance was very poor (high error rates and >50% *misbinding* or guess trials).

#### Stimuli

The stimuli were identical to those used in Experiments 1 and 2.

#### Task

**Figure 4 fig4:**
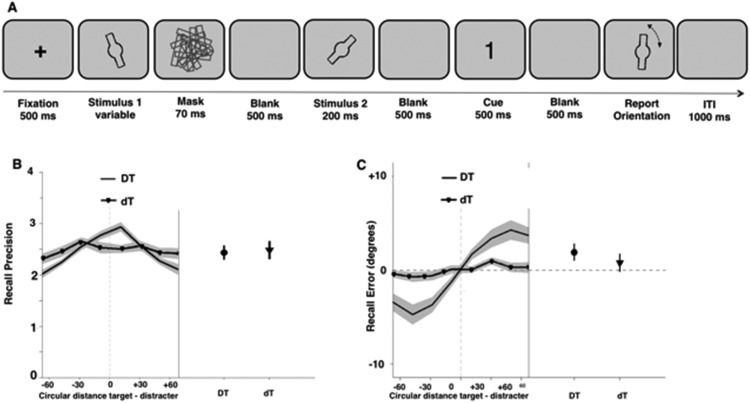
Design and results of the WM task used in Experiment 3. (A) A central fixation cross was presented for 500 ms followed by a first orientation stimulus, followed by a 70-ms mask. The presentation duration of the first orientation stimulus was varied to yield two conditions of equal probability: one in which the orientation was visible despite the mask and another in which the mask rendered the orientation stimulus invisible. After a 500-ms delay, the second orientation stimulus was presented for 200 ms (unmasked). This was followed by another 500-ms delay, and then by a 500-ms cue stimulus, which indicated to participants which orientation they had to report at the end of the trial. After a final 500-ms delay, participants reported the cued target orientation by matching the orientation of a probe stimulus with the cued orientation stimulus using the computer mouse. (B) Mean recall precision of cued orientation recall as a function of cued and uncued orientation similarity (left) and mean overall recall precision of cued orientation recall in Experiment 3 plotted for both conditions separately (*N* = 19). (C) Mean recall error as a function of cued and uncued orientation similarity in Experiment 3 plotted for the DT and dT condition separately (left), and mean overall recall error for the two conditions separately (*N* = 19).

As in Experiment 2, participants performed two tasks: a WM task and an identification task. The WM task (Figure 4A) was adapted from the task used in Experiment 2. The main difference was that participants were prompted to report the orientation of either the masked or unmasked stimulus. This was done to render the masked stimulus task-relevant, and allowed us to assess whether the orientation of the task-relevant subliminal stimulus could influence encoding of the unmasked item. Concurrently, this manipulation also allowed us to assess whether participants had any information available about the subliminal orientation when asked to reproduce it. The purpose of the identification task was to ensure that the pattern mask was effective at rendering stimuli subliminal.[Fig-anchor fig4]

Each trial began with the presentation of a central fixation cross for 500 ms followed by a first orientation stimulus, which was followed by a 70-ms mask. The presentation duration of the first orientation stimulus was varied to yield two conditions of equal probability: a first condition in which the orientation was visible despite the mask and another in which the mask rendered the orientation stimulus invisible (duration individually determined, *M* = 32 ms ± 6). After a 500-ms delay, the second orientation stimulus was presented for 200 ms (unmasked). This was followed by another 500-ms delay, and then by a 500-ms cue stimulus, which indicated to participants which orientation they had to report at the end of the trial. The cue was the number “1” or “2,” denoting the first or second orientation stimulus, respectively. After a final 500-ms delay, participants reported the cued target orientation by matching the orientation of a probe stimulus with the orientation of the cued stimulus using the computer mouse. Participants also completed an identification task that was identical to the one used in Experiment 2 (see supplementary materials for details).

#### Staircase procedure

The staircase procedure was the same as the one used in Experiment 2.

#### Design and procedure

Design and procedure were identical to Experiment 2 apart from the following changes. The WM task consisted of a fully factorial design with two variables: stimulus 1 visibility (subliminal, supraliminal) and cue (Stimulus 1, Stimulus 2). The different trial types were randomly intermixed within a block. Thus, the experimental design yielded four conditions: supraliminal distracter presented before a supraliminal target (we will refer to this condition as DT), subliminal distracter presented before a supraliminal target (dT), supraliminal target presented before presentation of a supraliminal distracter (TD), and subliminal target presented before a supraliminal distracter (tD).

Before starting the main experimental task, participants completed 50 practice trials in which they received feedback after every trial. Following practice, participants completed 24 blocks of 50 trials (1,200 trials in total) of the WM task, yielding 300 trials in each condition. The first 600 trials were completed in the first session and the last 600 trials in the second session. The identification task was identical to that in Experiment 2.

#### Analysis

##### Working-memory task

Effects on recall precision and recall error were tested using ANOVAs and *t* tests. First we tested whether task-relevant subliminal and supraliminal stimuli presented before the target can influence orientation reports. To that end we ran a 2 × 8 repeated-measures ANOVA with the variables “visibility” (subliminal, supraliminal) and “target-distracter similarity” (8 bins ranging from 90° clockwise from the target to 90° counterclockwise) on recall precision and recall error. In a second analysis, we examined whether a supraliminal distracter presented after the target can influence reports on the target using a one-way repeated-measures ANOVA with the variable “target-distracter similarity” on recall precision and recall error. Finally, we tested whether participants had any information available when asked to report a subliminal target presented before a supraliminal distracter. We reasoned that if participants had no orientation information available regarding the subliminal stimulus, the mean error should be high and not different from chance performance. We did not analyze recall precision, as in this case this measure might be contaminated: if a participant consistently reported the uncued, supraliminal orientation, recall precision would be high as errors in orientation report cluster around a certain value (the uncued orientation) and error variability is low in each bin. Recall error, on the other hand, would still be high and is therefore more informative about task performance.

##### Identification task

Performance in the identification task was assessed as described in Experiment 2.

### Results

#### Effects of subliminal and supraliminal distracters

##### Recall precision

Figure 4B shows the precision with which participants recalled the target orientation as a function of target-distracter similarity for the dT and the DT conditions. As in Experiment 2, the interaction between visibility and target-distracter similarity was significant (*F*(7, 140) = 4.18, *p* < .001, η^2^ = 0.173, JZS Bayes factor: 321). To examine this interaction further, two separate one-way repeated-measures ANOVAs assessed the effect of target-distracter similarity for each condition separately. These revealed a significant effect of target-distracter similarity on recall precision in the supraliminal (DT) condition (*F*(7, 140) = 9.09, *p* < .001, η^2^ = .313, JZS Bayes factor: >500) but not in the subliminal (dT) condition (*F*(7, 140) = 1.13, *p* = .347, JZS Bayes factor: 0.09). Specifically, the more similar the orientation of the supraliminal distracter was to the target orientation, the better was participants’ recall precision (see Figure 4B).

The main effect of target-distracter similarity on precision was also significant (*F*(7, 140) = 5.61, *p* < .001, η^2^ = 0.219, JZS Bayes factor: >500), but there was no main effect of visibility (*F*(1, 20) = .56, *ns*, JZS Bayes factor: 0.19).

##### Recall error

Figure 4C shows recall error for the target orientation as a function of target-distracter similarity for the dT and DT condition separately. As in the previous experiment, there was a significant interaction between visibility by target-distracter similarity (*F*(7, 140) = 5.49, *p* = .001, η^2^ = 1.507, JZS Bayes factor: > 500), reflecting a significant effect of target-distracter similarity in the supraliminal (DT) (*F*(7, 140) = 10.50, *p* < .001, η^2^ = 2.41, JZS Bayes factor: >500) but not the subliminal (dT) (*F*(7, 140) = 1.21, *p* = .310, JZS Bayes factor: 0.12) condition. When the supraliminal distracter orientation was counterclockwise to the target orientation (a negative circular distance in Figure 4C) there was a counterclockwise (negative) shift in participants’ orientation reports of the target. Similarly, when the supraliminal distracter orientation was clockwise to the target orientation (a positive circular distance in Figure 4C) there was a clockwise (positive) shift in participants’ orientation reports of the target. Subliminal distracters did not influence WM recall.

Analysis of performance in the identification task revealed that one participant was able to discriminate between stimulus present- and absent-trials, with the individual subjective ratings mirroring this pattern of performance. We reran the analysis excluding this participant (*N* = 20) and the results remained equivalent. Thus, we replicated the findings of the previous experiment that participants’ orientation reports are systematically shifted toward the distracter orientation if and only if the distracter is visible.

Our main interest was the influence of subliminal and supraliminal stimuli on WM encoding, but for completeness we also report results from our other two conditions (tD and and TD) in the supplementary materials.

##### Control analyses

To ensure that the systematic shifts in recall error with distracter orientation observed in Experiment 3 were not exclusively driven by trials in which participants incorrectly responded based on distracter stimuli (misbinding trials), we applied mixture-modeling to our data set, and excluded misbinding and guess trials. We then rerun our main analyses on recall precision and recall error. The same effects were observed (significant effect of target-distracter similarity in Condition DT but not Condition dT: *F*(7, 140) = 8.60, *p* < .001, ι^2^ = 2.105, JZS Bayes factor: >500; and *F*(7, 140) = 0.85, *p* = .518, JZS Bayes factor: 0.05, respectively).

#### Identification task

Performance in the prime identification task showed that, at the group level, participants were at chance at performing the discrimination task for the masked stimulus (see supplementary materials).

### Discussion

Experiment 3 replicated and extended the findings of Experiment 2: a distracter orientation influenced observers’ report of a remembered target orientation, whether it was presented before or after the target orientation. Observers’ reports of the target orientation were biased toward the distracter orientation. In contrast, the orientation of subliminal distracters did not influence responses. This was the case even when the distracter orientation was relevant for task completion—previously suggested to be a necessary condition for subliminal information to influence subsequent perception (Gayet et al., 2014).

## General Discussion

We have shown, using a novel and sensitive approach to measure influence of irrelevant stimuli on targets, that irrelevant distracters can influence WM performance to target stimuli when distracters are supraliminal but not when they are subliminal according to objective visibility measures. Experiments 1a and 1b replicated effects of subliminal stimuli on perceptual judgments (Eimer & Schlaghecken, 1998, 2002), while Experiment 2 demonstrated that these effects do not extend to WM decisions. Experiment 3 replicated the effects in Experiment 2 even when the subliminal stimuli were made task relevant. Interestingly, it also revealed that a supraliminal distracter orientation influences memory recall for a target orientation both when it appears before and when it appears after the target orientation. Whether this effect represents an encoding, response or maintenance effect is difficult to determine with the current set of results, and we leave this open to interpretation.

The present finding on subliminal stimuli and WM contrasts with findings by Silvanto and Soto (2012), who reported an effect of a subliminal distracter on WM representations of a target item. Participants retained a target orientation in memory to compare against a probe orientation stimulus. During the delay period, a masked subliminal distracter was presented on most trials, which was either congruent or incongruent with the target orientation. In the incongruent condition, but not the congruent or no-distracter conditions, participants’ accuracy was significantly impaired. A critical factor here, however, may be the level of awareness that participants had on the masked distracter. Crucially,  Silvanto and Soto (2012) defined levels of awareness based on subjective measures; a stimulus was classified as subliminal when observers reported not having seen it. We, on the contrary, classified stimuli as subliminal by presenting them in such a way that observers could not identify them above chance. Thus, our study defined stimuli as subliminal on the basis of an objective measure, while Silvanto and Soto (2012) used a subjective measure. The difference in results suggests that information of which observers are subjectively not aware can influence WM representations as long as observers show some sensitivity (*d*′ > 0) to the stimulus. However, there is no evidence of infiltration of WM for stimuli that are objectively below a threshold for awareness. The current results are consistent with the notion that items should be available for awareness to be encoded, or to influence, other WM representations (cf., Baddeley, 1986; Bundesen, 1990). The results may further suggest that WM is not simply a matter of activating sensory codes (cf., Cowan, 1995), regardless of their level of awareness.

It is important to remember that the notion of subliminal processing remains controversial (Pratte & Rouder, 2009; Reingold, 2004). Our two replications of the influence of subliminal primes on speeded responses in a visuomotor task similar to that used by Eimer and Schlaghecken (1998, 2002) add support to the possibility of subliminal effects, at least in the context of influencing motor tendencies in perceptual tasks. Our aim, in Experiments 2 and 3, was to test for putative effects of subliminal stimuli in the context of WM, but these experiments also differed from those in Experiment 1 in terms of the types of representations required to guide responses.

Indeed, an alternative explanation of the present set of results might focus less on the distinction between WM and perception, but instead on the nature of representations that observers need to access to complete the task. Notably, in Experiments 1a and 1a we implemented a strict response deadline and only stimulus-response assignments needed to be accessed. In Experiments 2 and 3, on the contrary, the perceptual content of the stimulus needed to be retrieved as observers had to report the visual appearance of a stimulus. Thus, it might be that subliminal effects are not readily observed in WM-type tasks because decisions require consideration of the appearance of an item, whereas observers’ responses can be modulated by subliminal information in tasks that require access to stimulus-response assignments. Future research could address this possibility by measuring subliminal effects in perceptual tasks that require access to the perceptual content of the stimuli, and subliminal effects in WM tasks that do not require access to it.

Mutual influences between supraliminal stimuli on observers’ reports have recently been reported in tasks using spatial frequency (Dubé, Zhou, Kahana, & Sekuler, 2014; Huang & Sekuler, 2010) and orientation stimuli (Fischer & Whitney, 2014). Huang and Sekuler (2010) asked participants to reproduce the spatial frequency of one of two successive Gabors, as indicated by a cue stimulus presented after the Gabors. The reproduced spatial frequency of the target item was influenced by the spatial frequency of the nontarget stimulus, and responses were biased in the direction of the nontarget item. This effect was found both within a trial, between a target and distracter stimulus, and between trials, from one target item to the subsequent target item. Similarly, Fischer and Whitney (2014) found a systematic bias in observers’ perceived orientations in the direction of previously seen orientations. Thus, this biasing effect appears to be robust across different experimental set ups and stimuli, and seems to be a general principle of information processing.

Importantly, here we explicitly controlled for the possibility that responses incorrectly based on the wrong stimulus may contaminate results and drive such biasing effects. The studies reviewed above did not model “misbinding.” What appears to be a biasing effect in the direction of the distracter might emerge when observers mistakenly report the distracter instead of the target on some small number of trials. Our analyses modeling and excluding misbinding and guessing trials unambiguously confirm a real influence of distracter stimuli on the memory for the target.

Unlike in perceptual and visuomotor processes, these WM biases were not induced by subliminal information. Gayet et al. (2014) suggested that subliminal information is only used when the context in which it is presented provides an incentive to make use of this information. However, we did not observe an effect of subliminal distracters on target recall even when observers were asked to report back the distracter’s orientation on some trials. Instead, we argue that WM representations are not as easily influenced by subliminal material and task-relevance may have a stronger mediating effect on the processing of subliminal stimuli in perceptual tasks, than in WM tasks such as ours.

We show an effect of a distracter on memory representations for a target item over and above misbinding in two separate experiments. This biasing effect was observed even though only two items (the target and the distracter) were presented, which is well below capacity limitations of WM. This finding suggests a permeability of memory representations even before capacity is exceeded, constraining current models of WM.

## Supplementary Material

10.1037/xhp0000052.supp

## References

[c1] Ansorge U., & Neumann O. (2005). Intentions determine the effect of invisible metacontrast-masked primes: Evidence for top-down contingencies in a peripheral cuing task. Journal of Experimental Psychology: Human Perception and Performance, 31, 762–777. 10.1037/0096-1523.31.4.762 16131248

[c3] Baddeley A. D. (1986). Working memory. New York, NY: Oxford University Press.

[c4] Bays P. M., Catalao R. F. G., & Husain M. (2009). The precision of visual working memory is set by allocation of a shared resource. Journal of Vision, 9, 1–11. 10.1167/9.10.7 19810788 PMC3118422

[c6] Brainard D. H. (1997). The Psychophysics Toolbox. Spatial Vision, 10, 433–436. 10.1163/156856897X00357 9176952

[c7] Bundesen C. (1990). A theory of visual attention. Psychological Review, 97, 523–547. 10.1037/0033-295X.97.4.523 2247540

[c8] Cowan N. (1995). Attention and memory: An integrated framework. (Oxford Psychology Series, No. 26). New York, NY: Oxford University Press.

[c9] Curtis C. E., & D’Esposito M. (2003). Persistent activity in the prefrontal cortex during working memory. Trends in Cognitive Sciences, 7, 415–423. 10.1016/S1364-6613(03)00197-9 12963473

[c10] Dehaene S., Naccache L., Le Clec’H G., Koechlin E., Mueller M., Dehaene-Lambertz G., . . . Le Bihan D. (1998). Imaging unconscious semantic priming. Nature, 395, 597–600. 10.1038/26967 9783584

[c11] Dienes Z. (2011). Bayesian versus orthodox statistics: Which side are you on? Perspectives on Psychological Science, 6, 274–290. Advance online publication 10.1177/1745691611406920 26168518

[c12] Dubé C., Zhou F., Kahana M. J., & Sekuler R. (2014). Similarity-based distortion of visual short-term memory is due to perceptual averaging. Vision Research, 96, 8–16. 10.1016/j.visres.2013.12.016 24395020 PMC4013795

[c14] Eimer M., & Schlaghecken F. (1998). Effects of masked stimuli on motor activation: Behavioral and electrophysiological evidence. Journal of Experimental Psychology: Human Perception and Performance, 24, 1737–1747. 10.1037/0096-1523.24.6.1737 9861720

[c15] Eimer M., & Schlaghecken F. (2002). Links between conscious awareness and response inhibition: Evidence from masked priming. Psychonomic Bulletin & Review, 9, 514–520. 10.3758/BF03196307 12412891

[c16] Eimer M., & Schlaghecken F. (2003). Response facilitation and inhibition in subliminal priming. Biological Psychology, 64, 7–26. 10.1016/S0301-0511(03)00100-5 14602353

[c101] Fischer J., & Whitney D. (2014). Serial dependence in visual perception. Nature Neuroscience, 17, 738–743. 10.1038/nn.3689 PMC4012025 24686785

[c17] Fisher N. I. (1993). Statistical analysis of circular data. New York, NY: Cambridge University Press 10.1017/CBO9780511564345

[c19] Gayet S., Van der Stigchel S., & Paffen C. L. E. (2014). Seeing is believing: Utilization of subliminal symbols requires a visible relevant context. Attention, Perception, & Psychophysics, 76, 489–507. 10.3758/s13414-013-0580-4 24186208

[c20] Gorgoraptis N., Catalao R. F. G., Bays P. M., & Husain M. (2011). Dynamic updating of working memory resources for visual objects. The Journal of Neuroscience, 31, 8502–8511. 10.1523/JNEUROSCI.0208-11.2011 21653854 PMC3124758

[c21] Greenwald A. G., Draine S. C., & Abrams R. L. (1996). Three cognitive markers of unconscious semantic activation. Science, 273, 1699–1702. 10.1126/science.273.5282.1699 8781230

[c102] Hannula D. E., Simons D. J., & Cohen N. J. (2005). Imaging implicit perception: Promise and pitfalls. Nature Reviews Neuroscience, 6, 247–255. 10.1038/nrn1630 15738960

[c22] Huang J., & Sekuler R. (2010). Distortions in recall from visual memory: Two classes of attractors at work. Journal of Vision, 10, 1–27. Advance online publication 10.1167/10.2.24 PMC4104522 20462325

[c23] Kiefer M., Ansorge U., Haynes J. D., Hamker F., Mattler U., Verleger R., & Niedeggen M. (2011). Neuro-cognitive mechanisms of conscious and unconscious visual perception: From a plethora of phenomena to general principles. Advances in Cognitive Psychology, 7, 55–67. 10.2478/v10053-008-0090-4 22253669 PMC3259028

[c24] Kleiner M., Brainard D., & Pelli D. (2007). What’s new in PsychToolbox-3? [ECVP Abstract Suppl..]. Perception, 36, 1.

[c25] Kouider S., & Dehaene S. (2007). Levels of processing during non-conscious perception: A critical review of visual masking. Philosophical Transactions of the Royal Society of London Series B, Biological Sciences, 362, 857–875. 10.1098/rstb.2007.2093 PMC2430002 17403642

[c27] Macmillan N. A., & Creelman C. D. (1991). Detection theory: A user’s guide. New York, NY: Cambridge University Press.

[c30] Neumann O., & Klotz W. (1994). Motor responses to nonreportable, masked stimuli: Where is the limit of direct parameter specification In Umilta C. & Moscovitch M. (Eds.), Attention and Performance XV (pp. 123–150). Cambridge, MA: MIT Press.

[c33] Pelli D. G. (1997). The VideoToolbox software for visual psychophysics: Transforming numbers into movies. Spatial Vision, 10, 437–442. 10.1163/156856897X00366 9176953

[c34] Pratte M. S., & Rouder J. N. (2009). A task-difficulty artifact in subliminal priming. Attention, Perception, & Psychophysics, 71, 1276–1283. 10.3758/APP.71.6.1276 19633343

[c35] Ramsøy T. Z., & Overgaard M. (2004). Introspection and subliminal perception. Phenomenology and the Cognitive Sciences, 3, 1–23. 10.1023/B:PHEN.0000041900.30172.e8

[c36] Reingold E. M. (2004). Unconscious perception: Assumptions and interpretive difficulties. Consciousness and Cognition: An International Journal, 13, 117–122. 10.1016/j.concog.2003.11.002 14990246

[c37] Silvanto J., & Soto D. (2012). Causal evidence for subliminal percept-to-memory interference in early visual cortex. NeuroImage, 59, 840–845. 10.1016/j.neuroimage.2011.07.062 21839180

[c40] Wyart V., Nobre A. C., & Summerfield C. (2012). Dissociable prior influences of signal probability and relevance on visual contrast sensitivity. PNAS Proceedings of the National Academy of Sciences of the United States of America, 109, 3593–3598. 10.1073/pnas.1120118109 PMC3295248 22331901

[c41] Zhang W., & Luck S. J. (2008). Discrete fixed-resolution representations in visual working memory. Nature, 453, 233–235. 10.1038/nature06860 18385672 PMC2588137

[c42] Zokaei N., Manohar S., Husain M., & Feredoes E. (2014). Causal evidence for a privileged working memory state in early visual cortex. The Journal of Neuroscience, 34, 158–162. 10.1523/JNEUROSCI.2899-13.2014 24381277 PMC3866482

