# Supplementary material for: Supraliminal But Not Subliminal Distracters Bias Working Memory Recall
Source: J Exp Psychol Hum Percept Perform. 2015 Apr 13;41(3):826–39. doi: 10.1037/xhp0000052 (PMC4445384; doi:10.1037/xhp0000052)
Supplement: Supplementary file 1 [file zfn003153236sf01.doc]

**Supplemental Materials**

**Supraliminal But Not Subliminal Distracters Bias Working Memory Recall**

**by T. Wildegger et al., 2015, *JEP: Human Perception and Performance***

**http://dx.doi.org/10.1037/xhp0000052**

**Supplementary Experimental Procedures**

*Experiment 2 and 3*

The prime identification task was identical to the task used in Experiment 1, except that only the dense-mask, but not the sparse-mask, condition was included (see Figure S1A). The probe stimulus was of the same orientation as the stimulus, or an orientation changed by 90° to the left or right (equally likely).
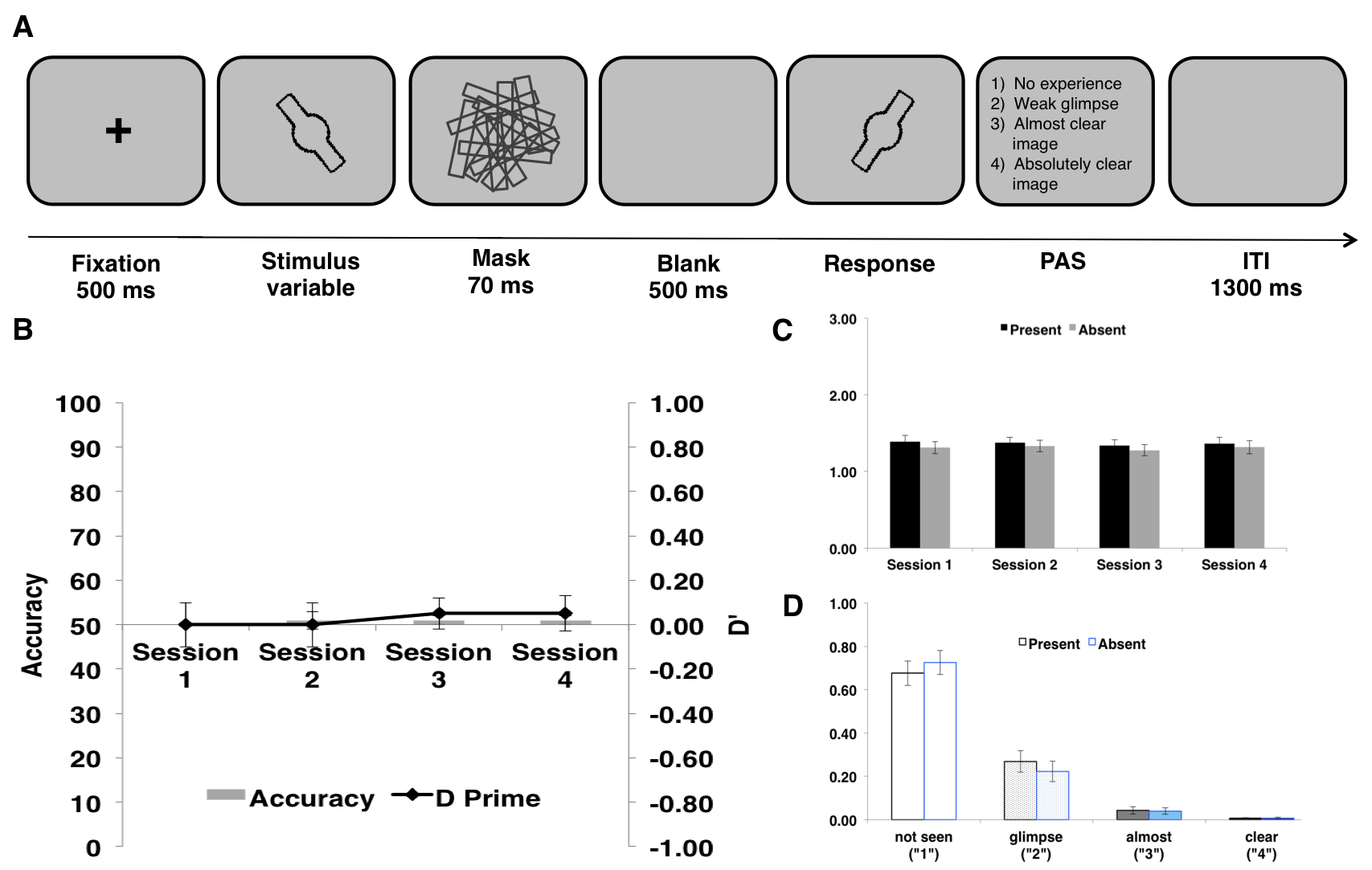


***Figure S1*.** Design of the prime identification task used in Experiments 2 and 3, and results of prime identification task in Experiment 2. **A**, The task sequence in the prime identification task was identical to Experiment 1 with the exception that the dense-mask, but not the sparse-mask, condition was included. **B**, Accuracy and d’ in the forced-choiced identification task for stimulus present condition as a function of session number in Experiment 2. Error bars reflect ± 1 standard error of the mean. **C**, Mean awareness ratings for the stimulus present and stimulus absent condition in Experiment 2 plotted for each session separately. **D**, Proportion of awareness ratings in the identification task for stimulus-present and stimulus-absent condition.

**Supplementary Results**

*Experiment 1b*

Prime Identification Task

*Accuracy and d’:* A paired-samples *t*-test compared accuracy in stimulus-present conditions using dense versus sparse masks. There was a significant difference between the conditions reflecting that participants performed significantly better in the sparse mask condition than in the dense-mask condition (*t*(19) = - 4.67, *p* < .001, *d* = 1.04, M_sparse_ = 65% ± 4, M_dense_ = 49% ± 2). Furthermore, performance in the dense-mask condition was not significantly better than chance (*t*(19) = - .39, *ns*).

Similarly, d’ in the sparse-mask and dense-mask conditions differed significantly (*t*(19) = -3.54, *p* <.01, *d* = 0.79). Participants performed significantly better in the sparse-mask than in the dense-mask condition (M_sparse_ = 1.03 ± 0.33, M_dense_ = - .04 ± .11). Importantly, d’ in the dense-mask condition was not significantly different from 0 (*t*(19) = .68, *ns*). These results show that, at the group level, participants were not better than chance at detecting and discriminating orientation in the dense-mask condition. However, stimuli followed by the sparse mask were reliably detected and their orientation discriminated. At the individual subject level five participants performed significantly better than chance (significant binomial test on accuracy data at the .05 level), suggesting they could differentiate between present and absent trials to some extent.

*Subjective Ratings:* We also replicated the pattern of subjective ratings reported in Experiment 1a. A Wilcoxon signed-rank test comparing average awareness ratings in the sparse-mask, dense-mask, and absent conditions indicated that there was no difference in ratings between the absent (M = 1.46 ± 0.14) and dense-mask condition (M = 1.42 ± 0.13, *Z* = -0.48, *ns*). However, there was a significant difference between the average awareness ratings in the sparse-mask (M = 2.19 ± 0.15) and absent condition (*Z* = -3.77, *p* < .001), and the sparse-mask and dense-mask conditions (*Z* = -3.77, *p* < .001, respectively).

To examine what was driving these differences, we calculated the proportions of each rating for each participant separately, and then ran eight separate Wilcoxon Signed-ranks tests comparing the proportions of ratings between the absent- and sparse-mask condition, and the dense-mask and sparse-mask condition for each rating option separately. “Not Seen” ratings occurred significantly more often in the dense-mask and absent condition compared to the sparse-mask condition (*Z* = -3.73, *p* < .001 and *Z* = -3.92, *p* < .001, respectively). Conversely, “Weak Glimpse”, “Almost Clear” and “Absolutely Clear” ratings were made significantly more often in the sparse-mask condition compared to the absent and dense-mask condition (all *p*’s < 0.04).

Together these results suggest that participants were unable to discriminate stimulus orientations on dense-mask trials, and unable to differentiate reliably between dense-mask and stimulus absent trials. Stimulus orientation on sparse-mask trials, however, was reliably discriminated. When we excluded the 5 participants who performed better than chance at the identification task from analysis of subjective ratings the same pattern of results was observed.

*Experiment 2*

*Accuracy and d’:* Figure S1B shows accuracy and d’ in the forced-choice discrimination task for the stimulus-present condition as a function of task session. Overall accuracy in the forced-choice discrimination task in the stimulus present condition was not significantly different from chance level (50%): *t*(23) = 1.60, *ns*; M = 51% ± 8%). A repeated-measures ANOVA with the factor “task session” (‘1’, ‘2’, ‘3’, ‘4’) revealed that accuracy did not vary with the task session (*F*(3,69) = .243, *ns*). Similarly, d’ in the stimulus-present condition was not significantly different from zero (M = .04 ± .214, *t*(23) = .425, *ns*) and showed no effect of task session (*F*(3,69) = .048, *ns*). These results suggest that, at the group level, participants were not better than chance at performing the discrimination task for the masked stimulus. At the individual subject level one participant performed significantly better than chance (significant binomial test on accuracy data at the .05 level [*p* = .044]), suggesting they could differentiate between present and absent trials to some extent.

*Subjective Ratings:* A Wilcoxon signed-ranks test comparing average awareness ratings in the stimulus-present and stimulus-absent condition indicated that ratings were higher in the present condition (M = 1.368 ± 0.08) than in the absent condition (M = 1.316 ± 0.07), *Z* = -2.40, *p* = .016, see Figure S1C).

To examine further what was driving these differences, we calculated the proportion of each rating for each participant separately, and then ran four separate Wilcoxon signed-ranks tests comparing the proportions of ratings between the present and absent condition for each rating option. “Not Seen” ratings occurred significantly more often in the absent condition (M = 0.73 ± 0.06) than in the present condition (M = 0.68 ± 0.06), *Z* = -2.63, *p* = .01). Conversely, proportions of “Weak Glimpse” ratings were significantly higher in the present condition (M = 0.268 ± 0.05) than in the absent condition (M = 0.222 ± 0.05, *Z* = -2.64, *p* = .008). Proportions of “Almost Clear” and “Absolutely Clear” did not differ between the absent condition (M = 0.039 ± 0.02, and M = 0.01 ± 0.004, respectively) and the present condition (M = 0.04 ± 0.02, and M = 0.01 ± 0.003, respectively), all *p’*s > 0.5. Figure S1D shows the proportion of awareness ratings for the stimulus-present and stimulus-absent conditions separately.

Together, these results suggest that, at the group level, participants were unable to discriminate stimulus orientations on present trials, reinforcing the results obtained in the d’ and accuracy analyses. However, analysis of the subjective ratings suggests that participants could differentiate between stimulus-present and stimulus-absent trials to a small degree, since participants reported not seeing a stimulus significantly more often when there was no stimulus compared to when there was. Conversely, when a stimulus was present, participants reported perceiving a weak glimpse more often than when there was no stimulus.

Importantly, subjective ratings converged with objective performance in the forced-choice identification task where participants did not perform better than chance. The only significant differences between stimulus present and absent trials in subjective ratings was between “Not Seen” and “Weak Glimpse” ratings, which participants were instructed to use to describe perceptual experiences that were lacking any orientation information. Conversely, ratings of “Almost Clear” and “Absolutely Clear”, which participants had been instructed to use when they had experienced orientation information, did not differ between conditions. Thus, subjective ratings indicated that participants could detect, but not identify, stimulus presence, which is consistent with the forced-choice identification task measures.

*Experiment 3*

WM Task: tD and TD conditions

*Recall precision*: Figure S2A shows the precision with which participants recalled the target orientation as a function of target-distracter similarity for the TD condition. There was no significant effect of target-distracter similarity on recall precision in the TD condition (*F*(7,140) = 1.87, *p* = .210, *ɳ*^2^ = .11).

*Recall error*: Figure S2C shows recall error for the target orientation as a function of target-distracter similarity for the TD condition. Visual inspection of the graphs suggests that recall error varied as a function of target – distracter difference. As before, when the distracter orientation was to the right of the target orientation there was a rightwards shift in participant’s orientation reports of the target, and vice versa. Statistical analysis of the data supported this and there was a strong trend for an effect of target-distracter similarity (*F*(7,140) = 2.60, *p* = .057, *ɳ*^2^ = .805). This suggests that presentation of a supraliminal distracter can influence the report of a remembered orientation, irrespective of whether it is presented before or after target orientation. The biasing effect in the TD condition was equivalent to the biasing effect in the DT condition (*t*(20) = -1.00, *p* = .33).

Figure S2B shows mean recall error in the tD condition for each subject separately. The mean error in the tD condition was 89° (±0.9°), which was not significantly different from chance (i.e. 90°; *t*(20) = -1.29, *p* = .209). This corroborates the prime identification results and shows that stimuli remain subliminal in the actual task, where reports of subliminal stimuli are randomised.

**
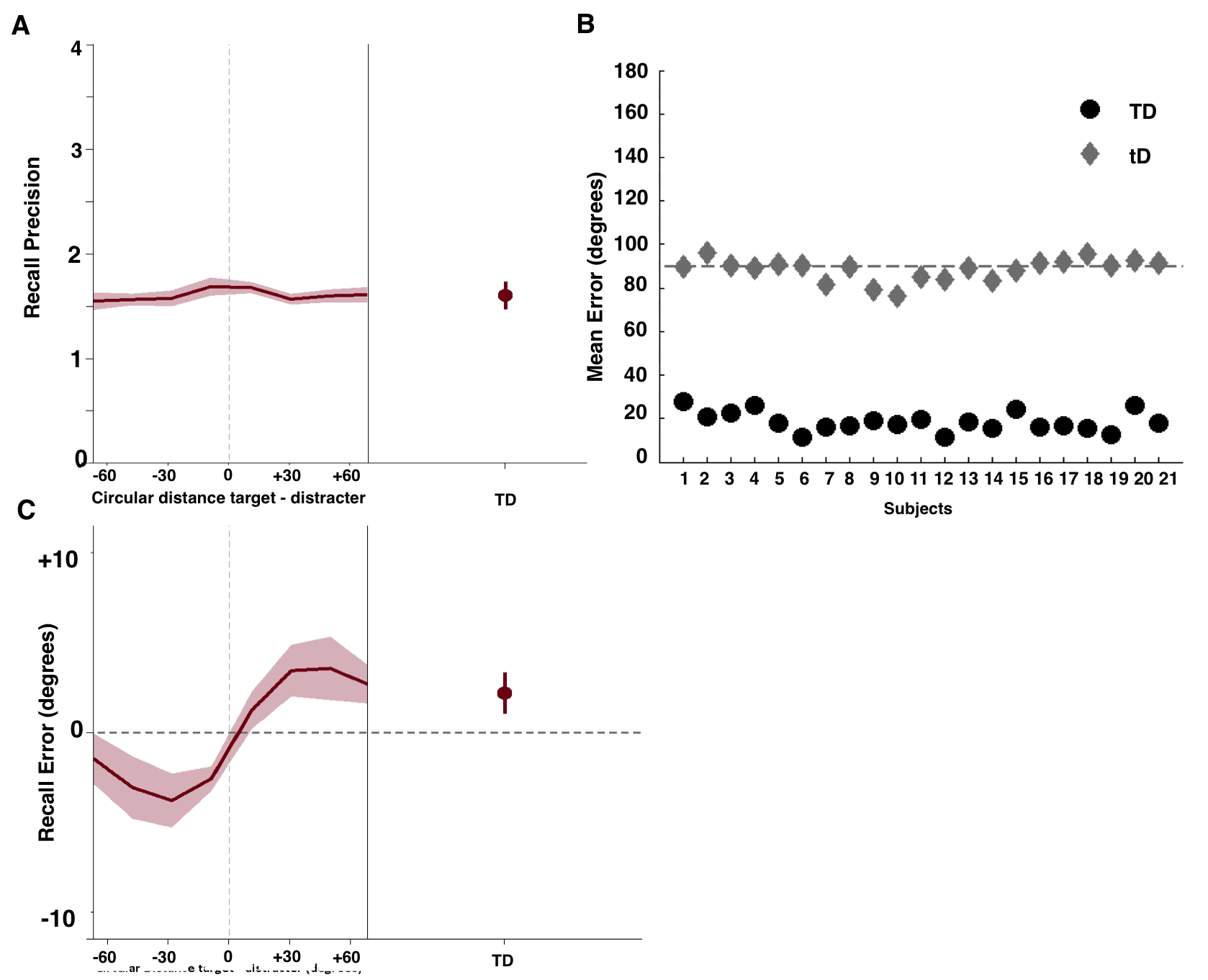
**

***Figure S2*.** **A**, Mean recall precision of cued orientation recall as a function of cued and uncued orientation similarity in Experiment 3 for the TD condition (left), and average recall precision of cued orientation recall for the same condition (N = 19). **B**, Mean error in reporting the cued orientation for the TD and tD conditions in Experiment 3 plotted for each subject separately. The dotted grey line indicates chance performance. **C**, Mean recall error as a function of cued and uncued orientation similarity for the TD condition (left), and mean overall recall error for the two conditions separately (N = 19).

*Control analysis*: To ensure that the systematic shifts in recall error with distracter orientation observed in Experiment 3 were not exclusively driven by trials in which participants incorrectly responded based on distracter stimuli (*misbinding* trials), we applied mixture-modelling to our data set, and excluded misbinding and guess trials. We then re-run our main analyses on recall precision and recall error. The qualitative pattern of results remained the same but the trend for a significant effect of target-distracter similarity in condition TD was now significant: *F*(7,140) = 3.02, *p* = .026, *ɳ*^2^= 0.918.

Prime Identification Task

Accuracy responses were not recorded during two of the four sessions for one participant due to technical errors and only the subjective ratings were included in the analysis. For another participant, both subjective ratings and accuracy responses from one session were not included in the analysis as they were accidentally overwritten.

*Accuracy and d’:* Figure S3A shows accuracy and d’ in the forced-choice discrimination task for the stimulus-present condition as a function of task session. Accuracy in the stimulus present condition was not significantly different from chance level (50%): *t*(18) = .35, *ns*; M = 50% ± 1%), and did not vary with the task session (F(3,54) = .73, *ns*). Similarly, d’ in the stimulus present condition was not significantly different from zero (*t*(18) = .37, *ns*; M = .02 ± .06). Performance did not vary over task blocks/sessions (*F*(3,54) = .96, *ns*). These results suggest that, at the group level, participants were not better than chance at performing the discrimination task for the masked stimulus. At the individual subject level, one participant performed significantly better than chance (significant binomial test on accuracy data at the .05 level [*p* < .001]), suggesting they could differentiate between present and absent trials to some extent.


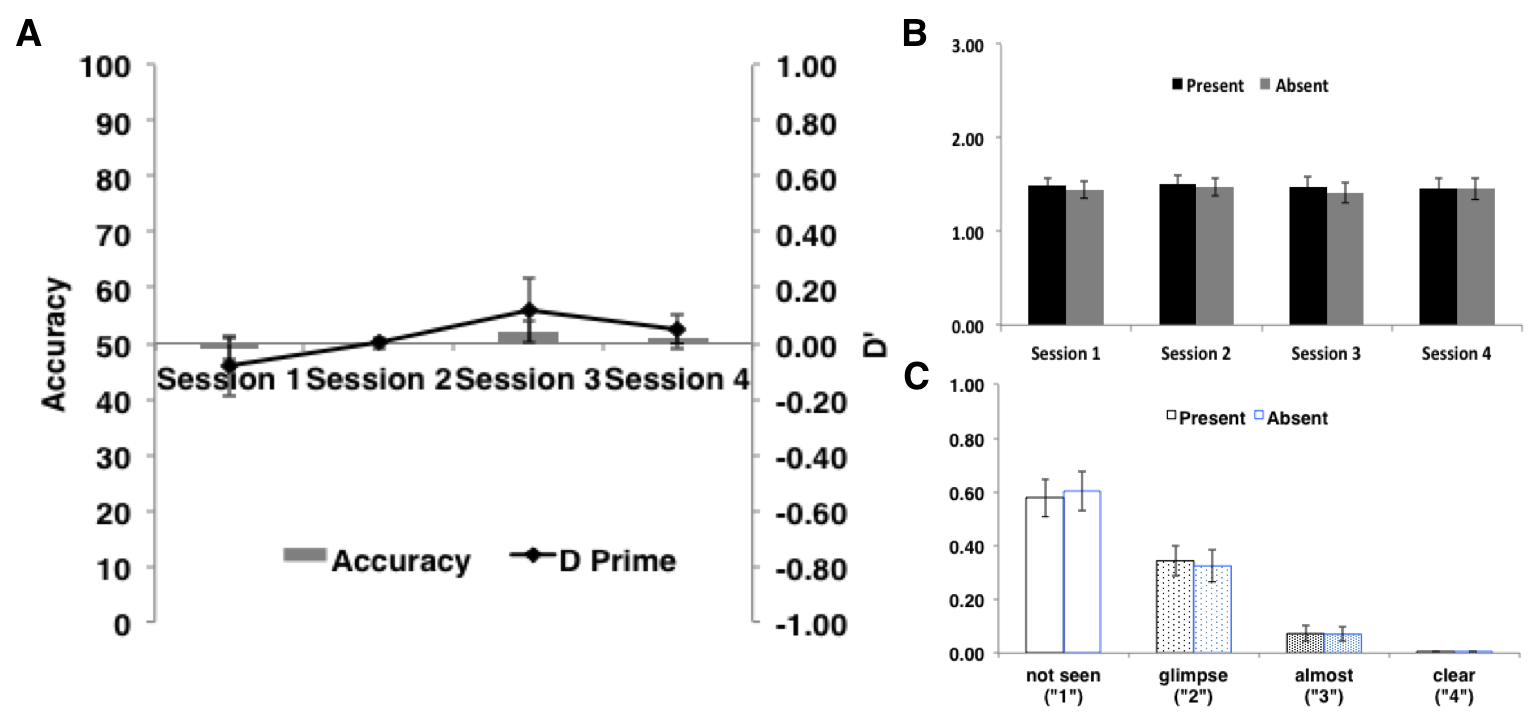


***Figure S3*.** **A**, Accuracy and d’ in the forced-choiced identification task for stimulus-present condition as a function of session number in Experiment 3. Error bars reflect ± 1 standard error of the mean. **B**, Mean awareness ratings for the stimulus present and stimulus absent condition in Experiment 3 for each session separately. **C**, Proportion of awareness ratings in the identification task for stimulus-present and stimulus-absent condition.

*Subjective Ratings.* Figure S3B shows the average awareness ratings for the stimulus present and absent conditions for each testing session. A Wilcoxon signed-ranks test comparing average awareness ratings in the stimulus-present and stimulus-absent condition indicated that overall ratings were not different between the present and absent condition (M = 1.45 ± 0.10; M = 1.48 ± 0.10, respectively). Furthermore, this did not change over the course of the experiment, and there was no difference between conditions in any of the sessions (all *Z*’s < 1).

Since there were no significant differences in average awareness ratings between stimulus-present and stimulus-absent conditions we did not statistically evaluate the proportions of different ratings given in each of the two conditions. However, for completeness the proportions of ratings are depicted in Figure S3C.

Together these results reinforce the results in the accuracy and d’ analyses, suggesting that participants were unable to discriminate stimulus orientations on present trials at the group level. Furthermore, analysis of the subjective ratings suggests that participants could not differentiate reliably between stimulus-present and stimulus-absent trials as participants.
